# Supplementary material for: Planning a future randomized clinical trial based on a network of relevant past trials
Source: Trials. 2018 Jul 11;19:365. doi: 10.1186/s13063-018-2740-2 (PMC6042258; doi:10.1186/s13063-018-2740-2)
Supplement: Supplementary file 1 — Planning a future randomized clinical trial based on a network of relevant past trials. (DOC 476 kb) [file 13063_2018_2740_MOESM1_ESM.doc]

# Planning a future clinical trial based on a network of relevant past trials

**Appendix**

Table of Contents

[1 Network of interventional strategies for in-stent restenosis 2](#__RefHeading___Toc487824048)

[1.1 Network meta-analysis of all trials for percent diameter re-stenosis 2](#__RefHeading___Toc487824049)

[1.2 Evidence obtained in 2013 3](#__RefHeading___Toc487824050)

[1.3 Studies published after 2013 3](#__RefHeading___Toc487824051)

[2 Network of biologics and triple therapy in methotrexate-naïve patients 4](#__RefHeading___Toc487824052)

[2.1 Data for ACR50 response 4](#__RefHeading___Toc487824053)

[2.2 Network meta-analysis 5](#__RefHeading___Toc487824054)

[2.3 Details and R code for the calculations of the conditional power for a ‘triple therapy versus methotrexate+etarnecept’ study; pairwise and network meta-analysis 6](#__RefHeading___Toc487824055)

[2.4 Power of a single trial designed as a standalone experiment 7](#__RefHeading___Toc487824056)

[2.5 Conditional power of an updated pairwise meta-analysis 8](#__RefHeading___Toc487824057)

[2.6 Conditional power of an updated network meta-analysis 8](#__RefHeading___Toc487824058)

[3 Description of methods to evaluate heterogeneity and incoherence 9](#__RefHeading___Toc487824496)

[3.1 Heterogeneity 9](#__RefHeading___Toc487824497)

[3.2 Incoherence 10](#__RefHeading___Toc487824498)

[4 Association between heterogeneity and precision 11](#__RefHeading___Toc487824590)

# Network of interventional strategies for in-stent restenosis

## Network meta-analysis of all trials for percent diameter re-stenosis

The data for this example are taken from Siontis et al. (1) and are also available in the GitHub repository.

This is what the data and network look like

## name year mean1 sd1 n1 mean2 sd2 n2
## 1 ARTIST 2002 55.7 19.9 146 63.6 22.3 152
## 2 RIBS 2003 46.0 23.0 226 45.0 25.0 224
## 3 Gamma-1 2001 53.2 20.5 121 45.6 25.9 131


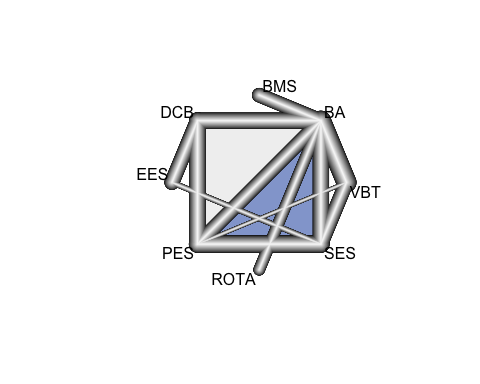


After running network meta-analysis (with the netmeta command) using all studies, we obtain the following results


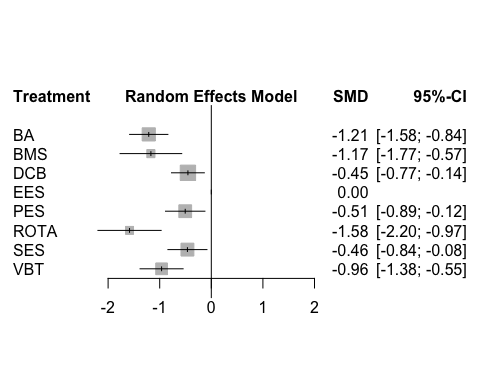


The standardized mean differences shown (SMD) are against everolimus-eluting stents (EES) which appear more effective in the treatment of in-stent restenosis than the widely used intervention with drug-coated balloons (DCB); SMD = -0.45 with 95%CI (-0.77; -0.14).

## Evidence obtained in 2013

Already in 2013, evidence from 19 trials favoured EES but the estimate was imprecise with wide confidence intervals and entirely based on indirect comparisons. Below are the results from the network meta-analysis had this taken place in 2013:


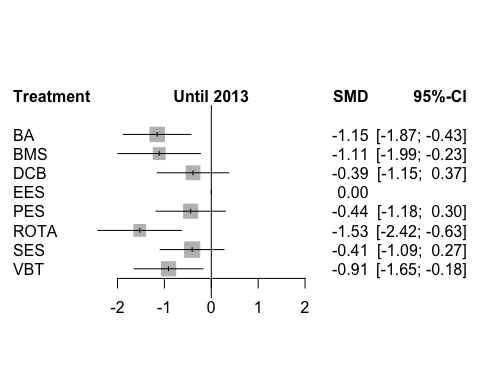


## Studies published after 2013

After 2013, two additional studies have been published that compare EES and DCB: -Alfonso F et al. A Prospective Randomized Trial of Drug-Eluting Balloons Versus Everolimus-Eluting Stents in Patients With In-Stent Restenosis of Drug-Eluting Stents: The RIBS IV Randomized Clinical Trial. J Am Coll Cardiol 2015;66:23–33.

-Alfonso F et al. A randomized comparison of drug-eluting balloon versus everolimus-eluting stent in patients with bare-metal stent-in-stent restenosis: the RIBS V Clinical Trial (Restenosis Intra-stent of Bare Metal Stents: paclitaxel-eluting balloon vs. everolimus-eluting stent). J Am Coll Cardiol 2014;63:1378–86.

Together they have a sample size of 489 patients.

We assumed that the new study is planned in populations similar to those examined in the RIBS IV and RIBS V studies cited above. The average standard deviation was 20. We assume a sought change in the percent diameter restenosis equal to -9% in favour of EES (equal to that identified by the NMA).

The estimated SMD in 2013 was -0.39 with 95%CI (-1.15, 0.37). A new study with SMD -0.45 and standard error 0.133, when added to the existing network would have resulted in a (pooled) SMD -0.43 with 95%CI (-0.86, 0).


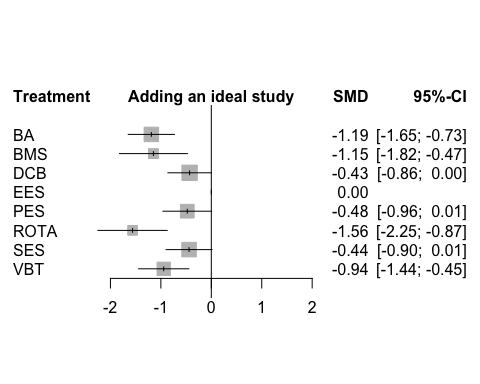


The sample size needed for a standard error 0.133 is 116 patients per arm; that is 232 randomized patients in total.

# Network of biologics and triple therapy in methotrexate-naïve patients

Here we explain in detail and using an R code how to run NMA and estimate conditional power.

## Data for ACR50 response

Data is as presented in the appendix in Hazlewood et al., BMJ 2016;353:i1777. Treatments are grouped in nodes as listed in the last column; the format of the data is as follows and data is also available in the GitHub repository. The data is named 'hazlewood'

head(hazlewood)

## id year study r1 n1 r2 n2 design t1 t2 y stderr
## 1 1 2015 AVERT 2015 54 116 75 119 E G E G 0.6714488 0.2659101
## 2 2 2009 AGREE 2009 107 253 147 256 E F E F 0.6098625 0.1793659
## 3 3 2014 HOPEFUL-I 2014 63 163 110 171 E H E H 1.0516420 0.2266216
## 4 4 2013 HITHARD 2013 41 85 56 87 E H E H 0.6619821 0.3118216
## 5 5 2013 OPTIMA 2013 176 517 268 515 E H E H 0.7429971 0.1280402
## 6 6 2008 Bejarano 2008 33 73 42 75 E H E H 0.4335339 0.3307803
## contrast
## 1 G - E
## 2 F - E
## 3 H - E
## 4 H - E
## 5 H - E
## 6 H - E

## Network meta-analysis

We run network meta-analysis using the netmeta command in netmeta package. Make sure you have installed and downloaded the package meta, netmeta and magic.

library(meta)sa
library(netmeta)
library(magic)

hazlenet<-netmeta(y,stderr,t1,t2,id,data=hazlewood, sm="OR",r="V",comb.fixed =F, comb.random = T)
summary(hazlenet)

## Number of studies: k = 29
## Number of treatments: n = 22
## Number of pairwise comparisons: m = 42
##
## Random effects model
##
## Treatment estimate (sm = 'OR', reference.group = 'V'):
## OR 95%-CI
## A 4.4586 [1.8708; 10.6258]
## B 1.3345 [0.7034; 2.5317]
## C 1.8427 [0.8223; 4.1293]
## D 1.3309 [0.3485; 5.0825]
## E 2.0631 [1.1310; 3.7634]
## F 1.1212 [0.5140; 2.4456]
## G 1.0542 [0.4418; 2.5155]
## H 0.9939 [0.5190; 1.9034]
## I 1.3802 [0.6444; 2.9563]
## J 1.1302 [0.4872; 2.6219]
## K 0.7113 [0.4185; 1.2090]
## L 1.5507 [0.6762; 3.5564]
## M 3.3923 [1.1516; 9.9927]
## N 1.0269 [0.4861; 2.1698]
## O 0.8626 [0.3940; 1.8885]
## P 3.0624 [1.4325; 6.5469]
## Q 1.2557 [0.5924; 2.6618]
## R 1.0936 [0.5158; 2.3184]
## S 0.6983 [0.2126; 2.2932]
## T 4.5703 [1.0180; 20.5179]
## U 1.3874 [0.6945; 2.7715]
## V . .
##
## Quantifying heterogeneity / inconsistency:
## tau^2 = 0.0321; I^2 = 34.3%
##
## Test of heterogeneity / inconsistency:
## Q d.f. p-value
## 21.31 14 0.0938

The treatment codes above are as follows

## 1 A: CyA
## 2 B: ETN
## 3 C: IM/sc MTX
## 4 D: IM/sc MTX & CyA
## 5 E: MTX
## 6 F: MTX & ABAT(IV)
## 7 G: MTX & ABAT(sc)
## 8 H: MTX & ADA
## 9 I: MTX & CTZ
## 10 J: MTX & CyA
## 11 K: MTX & ETN
## 12 L: MTX & GOL
## 13 M: MTX & HCQ
## 14 N: MTX & IFX
## 15 O: MTX & RTX
## 16 P: MTX & SSZ
## 17 Q: MTX & TCZ 4 mg/kg
## 18 R: MTX & TCZ 8 mg/kg
## 19 S: MTX & TOFA
## 20 T: SSZ
## 21 U: TCZ
## 22 V: Triple Therapy

We are interested in the relative effect of V versus K; here estimated OR=0.71 with 95% CI (0.42, 1.21).

## Details and R code for the calculations of the conditional power for a ‘triple therapy versus methotrexate+etarnecept’ study; pairwise and network meta-analysis

To estimate the required sample size for a new ‘triple therapy versus methotrexate+etanercept’ trial, we calculated the power of a single trial, the conditional power of an updated pairwise meta-analysis and the conditional power of an updated network meta-analysis. The estimated OR from the existing network meta-analysis was 0.71 with 95% CI (0.42, 1.21). We assumed a sought OR of 0.71 in ACR50 response favouring methotrexate + etanercept, equal to the respective random-effects network meta-analysis treatment effect. In all analyses, we assumed that the event rate in the triple therapy was equal to the average observed in the network (49%). The outcome was measured in the OR scale. Existing evidence is analysed using the fixed-effect model for the pairwise meta-analysis (as only one head-to-head study is available) and the random-effects model for the network meta-analysis. Heterogeneity was low to moderate (τ2=0.03) in the existing network. Future studies are assumed not to introduce further heterogeneity both in pairwise and in network meta-analysis. The sample size needed for a new ‘triple therapy versus methotrexate+etanercept’ trial based on network meta-analysis, pairwise meta-analysis and as a standalone experiment are 292, 790 and 1086 respectively. The R code for performing these calculations is given below. The functions cp.pma, cp.nma.setup and cp.nma.plot are required and can be found in <https://github.com/esm-ispm-unibe-ch>. A brief description of their functionality is given below

**cp.pma:** This function estimates the conditional power of an updated pairwise meta-analysis under a fixed or random effects model. The calculations are as in Roloff et al. (11).

**cp.nma.setup:** This function sets the data in the required format for calculating the conditional power of an updated network meta-analysis. An object of class cp.nma.setup is returned.

**cp.nma:** This function calculates the conditional power in a network meta-analysis for a comparison of interest. An object of class cp.nma.setup is required as input. The formulae used to calculate the power is given in Nikolakopoulou et al. (3) (equation 3 updated for random effects as described in section 3.3).

You can install the commands from GitHub.

install_github("esm-ispm-unibe-ch/ConditionalPower")

library(ConditionalPower)

The R packages **meta, netmeta** and **magic** also need to be installed.

## Power of a single trial designed as a standalone experiment

We estimate the power of a ‘triple therapy versus methotrexate+etanercept’ trial on its own using standard methods. Assuming a success rate of 0.49 and an OR=0.71 we calculate the sample size for a two-arm trial with 1:1 randomisation.

pC=0.49
OR=0.71
oddsT=pC/(OR*(1-pC))
pT=oddsT/(1+oddsT)
kappa<-1 #allocation ratio 1:1
alpha<-0.05 #type I error

PowerRCT<-c()
for (i in 1000:1200){
 nT=i/2
 z=log(OR)*sqrt(nT)/sqrt(1/(kappa*pC*(1-pC))+1/(pT*(1-pT)))
 PowerRCT=c(PowerRCT,pnorm(z-qnorm(1-alpha/2))+pnorm(-z-qnorm(1-alpha/2)))
}

print(min(c(1000:1200)[PowerRCT>=0.8]))

## [1] 1084

## Conditional power of an updated pairwise meta-analysis

We then estimate the conditional power of an updated network meta-analysis of the ‘triple therapy versus methotrexate+etanercept’ comparison. First we need to load the TEAR2012 study data; the data from the only direct study

## study year r1 n1 r2 n2
## 1 TEAR 2012 2012 91 244 41 132

Then we will use the command cp.pma using the following arguments:

data=the existing studies (here only TEAR 2012) level="arm"; specifies that we have arm-level data type="binary" ; type of the outcome effsize="OR" ; define the relative treatment effect measure, here OR p.c= the event rate; here we input the event rate in the control intervention (here set to 0.49)

alternative.ES= the sought difference between the groups (measured on the same scale as in the effect size); here OR=0.71 sample.size= the sample size in the new study; here set equal to 790.

Then, the function estimates the conditional power in an updated meta-analysis when one new study with a given sample size is added assuming 1:1 randomisation. Default type one error rate is 5%.

conditionalPowerPairwise=cp.pma(data=TEAR2012,level="arm",type="binary",effsize="OR",sample.size=790,p.c=0.49, alternative.ES=0.71 )
print(conditionalPowerPairwise$cp)

## [1] 0.8000667

This optimal sample has been found after trying all sample size values between 80 and 1200.

## Conditional power of an updated network meta-analysis

We then estimate the conditional power of an updated network meta-analysis of the ‘triple therapy versus methotrexate+etanercept’ comparison. We will use the data in a different format (per arms). The data are in hazlewoodperarm.csv.

## year study id t r n
## 1 2015 AVERT 2015 1 MTX 54 116
## 2 2015 AVERT 2015 1 MTX and ABAT(sc) 75 119
## 3 2009 AGREE 2009 2 MTX 107 253
## 4 2009 AGREE 2009 2 MTX and ABAT(IV) 147 256
## 5 2014 HOPEFUL-I 2014 3 MTX 63 163
## 6 2014 HOPEFUL-I 2014 3 MTX and ADA 110 171

Then, we need to transform the data into an cp.nma.setup object which is suitable for conditional power in NMA calculations.

hazlewoodCP<-cp.nma.setup(data=hazlewoodperarm, level="arm", type="binary", effsize="OR")

This object, here named hazlewoodCP, is taken as argument in the cp.nma function. An important item within the hazlewoodCP object is the comparisons; that lists all *possible* pairwise comparisons

head(hazlewoodCP$comparisons)

## [1] "CyA:ETN" "CyA:IM/sc MTX" "CyA:IM/sc MTX and CyA"
## [4] "CyA:MTX" "CyA:MTX and ABAT(IV)" "CyA:MTX and ABAT(sc)"

In the cp.nma function you need to define the targeted comparisons and the design of the future studies. At the moment the function assumes that the targeted comparison will be the one that we will try in a future study. The argument design takes a character defining the comparison between treatments and has to be one of the comparisons listed in the cp.nma.setup object. You need to consider the hazlewoodCP$comparisons vector to define the correct comparison. In our case, it is the 176th comparison.

hazlewoodCP$comparisons[176]

## [1] "MTX and ETN:triple"

In the argument sample.size.range you need to input one or more values of possible total sample size in a new two-arm study. The function then assumes an 1:1 randomisation. The other arguments are as in cp.pma function.

conditionalPowerNMA<-cp.nma(hazlewoodCP,design="MTX and ETN:triple",sample.size.range=280, p.c=0.49, alternative.ES=0.71)
print(conditionalPowerNMA)

## $sample.siz
## [1] 280
##
## $adding.study.comparing
## [1] "MTX and ETN:triple"
##
## $conditional.power
## [,1]
## [1,] 0.8008542

The sample size was estimated after trying values between 80 and 1200.

# Description of methods to evaluate heterogeneity and incoherence

## Heterogeneity

Over the last two decades the I^2^ measure for heterogeneity became very popular and substitute the Q-test for heterogeneity which has been shown to have poor statistical properties. I^2^ measures the percentage of variability due to heterogeneity rather than sampling variance and can be intuitively understood as the extend to which the confidence intervals of the study-specific effect sizes overlap (2). For this reason, meta-analyses of small studies often present low I^2^. Sensible interpretation of I^2^ requires consideration of its uncertainty conveyed by the 95% confidence interval.

Measuring the actual heterogeneity variance (the variance of the distribution of the random effects) is presented in the literature as a sensible way forward to infer about heterogeneity. The heterogeneity standard deviation is measured on the same scale as the outcome (e.g. on the mean difference scale or log odds ratio scale). Consequently, a comparison of the actual summary effect to the heterogeneity standard deviation can give a hint about the relative magnitude of the heterogeneity. However, recent empirical studies have produced tables of the ‘expected’ heterogeneity according to the type of treatment comparison (e.g. pharmacological intervention versus placebo) and outcome (e.g. mortality). The expected distribution of heterogeneity variance in a dichotomous outcome is presented in Table 4 in (3) and for continuous outcomes in Table 3 in (4).

## Incoherence

The assumption of transitivity is central to network meta-analysis and should be carefully considered. Important intransitivity can manifest in the data as incoherence; that is disagreement between direct and indirect evidence and diminish the validity of NMA results and conclusions. Methods to evaluate consistency can be grouped under local and global depending on whether they focus on parts of the evidence or evaluate the assumption in the entire network.

It is important to note that local methods for assessing incoherence suffer from low power; thus, it has been suggested that incoherence should be judged as statistical significant when p-value>0.10 (5,6). Global methods are expected to have higher power but this has not been yet formally evaluated.

*The loop-specific approach*

This is the simplest and the most commonly used method to assess incoherence locally (7,8).The absolute difference between the direct and the indirect treatment effects is estimated in each closed loop of evidence (often called incoherence factor
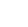
). A z-statistical test of the hypothesis of consistency can then be estimated 𝑧=
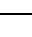
~𝑁
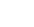
.

*Separating indirect and direct evidence*

In this method we contrast a direct treatment effect for a specific comparisons with the indirect treatment effect *from the entire network*. To obtain this ‘total’ indirect estimate we exclude the comparison of interest from the network and we re-perform the network meta-analysis. Their difference defines the comparison-specific incoherence factor, which again defines a z-test. The method was initially termed as “node splitting” and was sensitive to the parameterization of multi-arm trials (9).

*Global tests and models for incoherence*

Global approaches for assessing incoherence aim to test the entire network. This is done either by removing all consistency equations (unrelated mean effects model) or by allowing extra variability in the NMA model to account for differences between the various sources of evidence (9). Statistics that encompass heterogeneity and incoherence have been proposed as an alternative method to measure of the percentage of variability that cannot be attributed to random error or heterogeneity (10).

The *design by treatment* (DBT) interaction model is an network meta-analysis model where incoherence terms (IF) are added. The model results and a
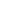
-test that tests jointly all incoherence terms for being close to zero.

# Association between heterogeneity and precision

For a pairwise meta-analysis the precision of the summary effect is


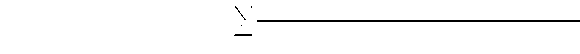


with an upper bound equal to the number of studies divided by the heterogeneity variance.

**References**

1. Siontis GCM, Stefanini GG, Mavridis D, Siontis KC, Alfonso F, Pérez-Vizcayno MJ, et al. Percutaneous coronary interventional strategies for treatment of in-stent restenosis: a network meta-analysis. Lancet Lond Engl. 2015 Aug 15;386(9994):655–64.

2. Higgins JPT, Thompson SG, Deeks JJ, Altman DG. Measuring incoherence in meta-analyses. BMJ. 2003 Sep 4;327(7414):557–60.

3. Turner RM, Spiegelhalter DJ, Smith GCS, Thompson SG. Bias modelling in evidence synthesis. J R Stat Soc Ser A Stat Soc. 2009 Jan;172(1):21–47.

4. Rhodes KM, Turner RM, Higgins JPT. Empirical evidence about incoherence among studies in a pair-wise meta-analysis. Res Synth Methods. 2015 Dec 17;

5. Veroniki AA, Vasiliadis HS, Higgins JPT, Salanti G. Evaluation of inconsistency in networks of interventions. Int J Epidemiol. 2013 Feb;42(1):332–45.

6. Veroniki AA, Mavridis D, Higgins JP, Salanti G. Characteristics of a loop of evidence that affect detection and estimation of inconsistency: a simulation study. BMC Med Res Methodol. 2014 Sep 19;14(1):106.

7. Bucher HC, Guyatt GH, Griffith LE, Walter SD. The results of direct and indirect treatment comparisons in meta-analysis of randomized controlled trials. J Clin Epidemiol. 1997 Jun;50(6):683–91.

8. Nikolakopoulou A, Chaimani A, Veroniki AA, Vasiliadis HS, Schmid CH, Salanti G. Characteristics of networks of interventions: a description of a database of 186 published networks. PloS One. 2014;9(1):e86754.

9. Dias S, Welton NJ, Sutton AJ, Caldwell DM, Lu G, Ades AE. Evidence synthesis for decision making 4: inconsistency in networks of evidence based on randomized controlled trials. Med Decis Mak Int J Soc Med Decis Mak. 2013 Jul;33(5):641–56.

10. Jackson D, Barrett JK, Rice S, White IR, Higgins JPT. A design-by-treatment interaction model for network meta-analysis with random inconsistency effects. Stat Med. 2014 Sep 20;33(21):3639–54.

11. Roloff V, Higgins JPT, Sutton AJ. Planning future studies based on the conditional power of a meta-analysis. Stat Med. 2013 Jan 15;32(1):11–24.
